# Possible Role of Metformin as an Immune Modulator in the Tumor Microenvironment of Ovarian Cancer

**DOI:** 10.3390/ijms22020867

**Published:** 2021-01-16

**Authors:** Faye K. Tsogas, Daniel Majerczyk, Peter C. Hart

**Affiliations:** 1 College of Science, Health and Pharmacy, Roosevelt University, Schaumburg, IL 60173, USA; ftsogas@mail.roosevelt.edu (F.K.T.); dmajerczyk@roosevelt.edu (D.M.); 2 Loyola Medicine, Berwyn, IL 60402, USA

**Keywords:** ovarian cancer, metformin, omentum, tumor microenvironment, T cell, myeloid-derived suppressor cell, neutrophil, macrophage

## Abstract

Growing evidence suggests that the immune component of the tumor microenvironment (TME) may be highly involved in the progression of high-grade serous ovarian cancer (HGSOC), as an immunosuppressive TME is associated with worse patient outcomes. Due to the poor prognosis of HGSOC, new therapeutic strategies targeting the TME may provide a potential path forward for preventing disease progression to improve patient survival. One such postulated approach is the repurposing of the type 2 diabetes medication, metformin, which has shown promise in reducing HGSOC tumor progression in retrospective epidemiological analyses and through numerous preclinical studies. Despite its potential utility in treating HGSOC, and that the immune TME is considered as a key factor in the disease’s progression, little data has definitively shown the ability of metformin to target this component of the TME. In this brief review, we provide a summary of the current understanding of the effects of metformin on leukocyte function in ovarian cancer and, coupled with data from other related disease states, posit the potential mechanisms by which the drug may enhance the anti-tumorigenic effects of immune cells to improve HGSOC patient survival.

## 1. Introduction

Among gynecologic carcinomas, high-grade serous ovarian cancer (HGSOC) is the highest cause of cancer-related mortality, bearing a poor survival rate of roughly 50% over five years due to the extremely metastatic nature of this disease [1,2,3]. Early detection is difficult since symptoms are nonspecific and the disease often presents in patients after the tumor has already metastasized within the abdominal cavity, which typically occurs in the commonly progressive HGSOC [2]. While optimal debulking of the tumor during initial surgery is associated with improved survival rates of HGSOC patients [4,5], typically, the widespread dissemination into the peritoneal cavity at the time of clinical presentation of HGSOC is marked by poorer survival due to incomplete cytoreduction, subsequent recurrence, and metastasis [2,6].

During metastasis, HGSOC tumor cells undergo the processes of adhesion, proliferation, migration, and invasion into the peritoneal cavity and preferentially latch onto the adipose tissue depot known as the omentum [6]. Many stromal cell types encompass the omentum and peritoneum, including mesothelial cells, fibroblasts, macrophages, T lymphocytes, and neutrophils [6,7]. Mesothelial cells superficially line the surface of the omentum, making them the initial target site during metastasis; however, other cells, such as fibroblasts and adipocytes, in the submesothelial space of the omentum and peritoneum have also been implicated in regulating HGSOC tumor cell adhesion and colonization of these tissues [6]. In addition to these numerous cell types present in this tumor microenvironment (TME), recent evidence suggests that immune cells play unique yet critical roles in the adhesion and colonization of the omentum and other intra-abdominal sites, which will be further discussed. As such, the stroma of the TME presents itself as an attractive, albeit complicated, target for the development of novel therapeutic strategies to complement cytoreductive surgery and chemotherapy (e.g., carbotaxol).

As the survival rates of patients with HGSOC have not dramatically improved over the last few decades [3], it is critical for novel therapeutic approaches for prevention and intervention of the disease to be developed in order to improve patient outcomes, such as overall and progression-free survival. Advances using novel compounds that selectively inhibit poly(ADP-ribose) polymerase (PARP), such as olaparib, have shown significant clinical efficacy against many subtypes of ovarian cancer (reviewed in [8,9]) and have recently been approved for treating recurrent epithelial ovarian cancer, among other related diseases; however, initial phase I clinical trials had occurred well over 10 years ago (reviewed in [10]), with PARP first being described decades prior [11]. While de novo drug development can bring significant improvements to patient care, it is generally exceptionally costly and can typically take upward of 15 years. An alternative approach is drug repurposing, in which a compound currently indicated for a disease is utilized for another disease, thus markedly increasing the rapidity of its clinical use through bypassing preclinical toxicity profiling and phase I clinical trials [12].

Metformin, a biguanide antidiabetic medication, is one such drug that has gained overwhelming attention in the treatment of inflammatory diseases, as well as a number of cancers. Early retrospective epidemiological case-control studies have indicated a possible association for metformin to enhance progression-free survival in patients with diabetes when compared to controls, which include patients with diabetes receiving other medications, as well as patients without diabetes [13,14]. Consistently, a subsequent meta-analysis of numerous retrospective studies showed a nearly twofold decrease in the risk of mortality across seven cohorts (cumulative odds ratio of 0.55), as well as a small but significant decrease in the incidence of ovarian cancer in several others [15]. Several prospective clinical trials have been initiated to determine the efficacy of metformin in the treatment of ovarian cancer, which are summarized in Supplementary Table S1, including one with recently published findings showing that metformin improved the overall survival and demonstrated a potential for the drug to promote platinum sensitivity in cancer stem cells ex vivo [16]. Interestingly, in another recently published prospective study, metformin was associated with decreased occurrence in adenomatous polyp reformation in colorectal cancer [17], further supporting its potential utility in preventing early tumor development and preventing tumor progression.

In ovarian cancer, in vivo xenograft studies have shown that clinically relevant doses of metformin given in a preventative regimen, including pretreatment prior to and maintenance during engraftment, have decreased the size of the primary tumor and inhibited the number of metastatic implants [18], suggesting that the physiological effects of the drug may involve its activity in the TME. In line with these findings, recent studies have demonstrated that the drug could also specifically target mesothelial cells in a 3D organotypic model of invasion of the omentum, which was consistent with the decreased adhesion of HGSOC tumor cells to the omentum in explants removed from patients on metformin when compared to matched controls [19]. Moreover, in co-culture systems, metformin was observed to also inhibit chemoresistance by inhibiting NF-κB-dependent IL-6 secretion from fibroblasts [20], as well as adipocyte-induced tumor cell proliferation and migration [21]. Together, these data suggest that metformin may have a significant effect on multiple stromal cell types in the TME of ovarian cancer. Unfortunately, despite the growing evidence that leukocytes also play a significant role in HGSOC tumor progression, limited data exist regarding the impact of metformin on immune cell recruitment and function in the TME. In this brief report, we will summarize the current data that indicate that metformin may target the immune cells of the TME of ovarian cancer and describe supporting evidence in other disease states that may give insight into the potential mechanisms by which metformin could regulate immune cell function to improve patient outcomes in HGSOC. 

## 2. Potential for Immunoregulation by Metformin in the Ovarian Tumor Microenvironment 

### 2.1. Metformin and T Cells—An Overview

The diverse activity of tumor-infiltrating lymphocytes (TILs) may play a significant role in the progression of many types of cancers, as an increased presence of cytotoxic CD8^+^ TILs (CTC) has often been associated with improved overall survival [22], whereas an increased frequency of immunosuppressive CD4^+^ regulatory T cells (Treg) in the TME correlates with poorer prognosis [23]. In general, this relates to an immune-resistant TME in which tumor cells evade cell-mediated apoptosis [24], allowing for unchecked proliferation, and with it, clonal expansion of cells that may possess profound self-renewal and invasive capacities. Early studies by Sato and colleagues evaluated the high levels of intraepithelial CD8^+^ TILs and CD4^+^/CD25^+^ Treg cells in the epithelium and stroma of several histologic subtypes of ovarian cancer (OvCa). Most T lymphocyte subtypes failed to demonstrate an increase in survival rate, except for the intraepithelial CD8^+^ TILs. Importantly, they found that a high intraepithelial CD8^+^/CD4^+^ TIL ratio was associated with an improvement in the survival rate, whereas CD4^+^ TILs alone did not. These findings demonstrated the influence of CD8^+^ TILs on the prognosis for OvCa. Similarly, intraepithelial CD4^+^/CD25^+^ Tregs alone failed to show an increase in survival rate, while a high CD8^+^/Treg (CTC/Treg) ratio was associated with improved survival of patients with ovarian cancer [25]. 

**Figure 1:**
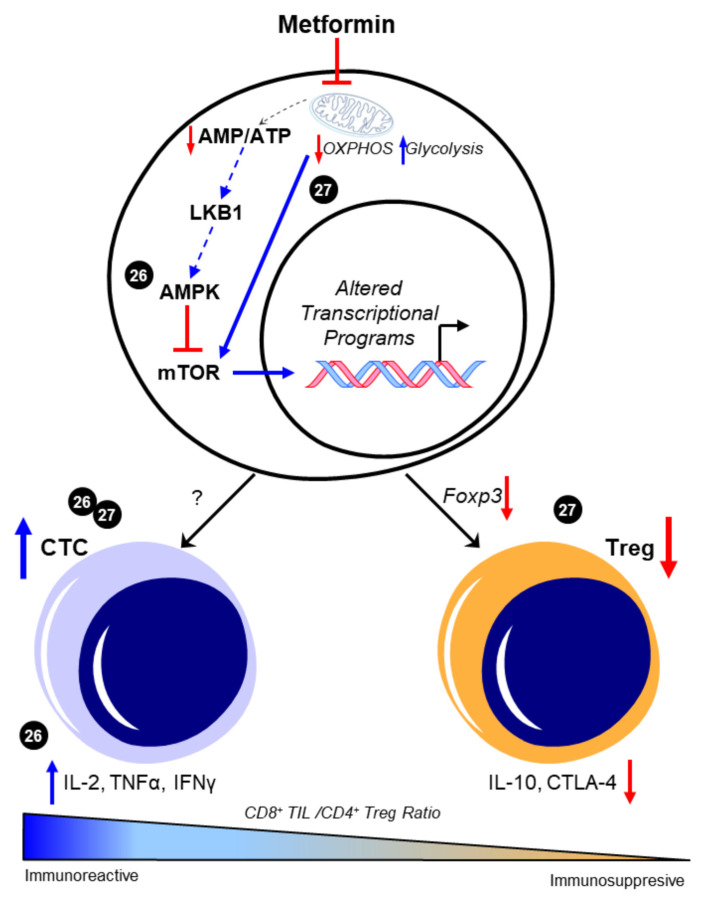
Metformin enhances CD8^+^/CD4^+^ ratio to favor an immunoreactive microenvironment. The canonical pathway of metformin intracellular activity that results in 5′-AMP-activated protein kinase (AMPK)-dependent inhibition of the mammalian target of rapamycin (mTOR) is shown. Presumably, this enhancement in AMPK and the consequent mTOR inhibition is attributed to the metabolic effects of metformin in promoting glycolysis through inhibited mitochondrial respiration; however, this was not experimentally shown in these studies and is represented by dotted lines. The ability of metformin to increase the CD8^+^/CD4^+^ ratio [26,27] was associated with increases in functional CD8^+^ tumor-infiltrating lymphocytes (TILs) and concomitant increases in interleukin 2 (IL-2), tumor necrosis factor alpha (TNFα), and interferon gamma (IFNγ) [26]. Decreases in Foxp3-driven CD4^+^ T cell phenotypes and the subsequent reduction in interleukin 10 (IL-10) and cytotoxic T lymphocyte antigen-4 (CTLA-4) expression were also separately observed [27]. Cited experiments are denoted by their respective reference numbers [26,27]. CTC: cytotoxic CD8^+^ TILs, OXPHOS: oxidative phosphorylation, Treg: regulatory T cells. Blue arrows indicate upregulation/activation, red arrows indicate downregulation/inactivation and red T symbols indicate direct inhibition. Solid lines represent data presented in the cited manuscript; dotted lines indicate informed interactions from well-established data.

Evidence supports the claim that CD8^+^ TIL function is of great importance in the prognosis and the survival rate in patients with HGSOC. To date, the ability to pharmacologically modulate cytotoxic T cell behavior in the metastatic TME of HGSOC has not yet been evaluated using preclinical models. However, several studies demonstrate the potential impact of metformin on maintaining CD8^+^ TIL function and possibly enriching a high CD8^+^/CD4^+^ ratio to promote an immunoreactive environment in other cancers. In a study by Eikawa et al. [26], metformin demonstrated inhibition of the growth of solid tumors in vivo in leukemia, melanoma, non-small-cell lung carcinoma, breast cancer, renal cancer, and intestinal cancer due to its ability to halt the exhaustion and apoptosis of CD8^+^ TILs in the TME. These findings were exemplified through the increase of three cytokines that the PD1^−^/Tim3^+^ phenotypic CD8^+^ TILs produce, consisting of interleukin-2 (IL-2), tumor necrosis factor alpha (TNFα), and interferon gamma (IFNγ). They also illustrated how metformin induced the differentiation from central memory T cells (Tmem) to effector memory T cells, demonstrating a stronger memory immune response to recurring tumor development. The authors attributed these findings in part to 5′-AMP-activated protein kinase (AMPK)-dependent inhibition of the mammalian target of rapamycin (mTOR) [26]. More recently, Kunisada et al. observed that metformin inhibited Treg induction and activity at the site of tumors in fibrosarcoma, leukemia, and melanoma in vitro by downregulating Foxp3, a transcription factor that promotes Treg phenotypes, thereby hindering the TGF-β-dependent differentiation of naïve CD4^+^/CD25^−^ T cells into Tregs [27]. Subsequent studies evaluated metformin’s effect on the ratio between tumor-infiltrating Tregs and tumor-infiltrating CD8^+^ T cells. When treated with metformin, an increase in CD8^+^ TILs was observed, suggesting that metformin promoted either increased recruitment or differentiation toward an antitumor T cell phenotype. Moreover, in vivo findings showed that metformin effectively delayed tumor growth by causing a shift from oxidative phosphorylation (OXPHOS) to glycolysis, which then decreased the Treg expression of interleukin-10 (IL-10) and cytotoxic T lymphocyte antigen-4 (CTLA-4) [27]. Intriguingly, the ability of metformin to alter CD4^+^ T cell phenotypes in vivo was associated with its activation of mTOR, as the mTOR inhibitor rapamycin ablated the ability of metformin to suppress Treg abundance. This mechanism is in contrast with the observed prevention of CD8^+^ TIL exhaustion resulting from the inhibition of mTOR by AMPK in the previous study by Eikawa et al. While convincing, it is contradictory to its canonical negative regulation of mTOR that was observed in most reports across disease states (reviewed in [28]). Taken together, these data suggest that metformin may have unique effects on specific subtypes of T cells, possibly due to their distinctive metabolic features [29], as well as their markedly different proteomic responses to external stimuli [30]. However, both indicate that metformin may favor an immunoreactive distribution of T cell phenotypes. The potential regulation of T cell differentiation and the subsequent CTC/Treg ratio by metformin is depicted in Figure 1.

### 2.2. Metformin and T Cells—Metabolic Targets and T Cell Phenotypes

While the molecular mechanisms underlying metformin’s potential enhancement of T cell function in the TME are still poorly understood, it is clear that its primary intracellular target upstream of AMPK, liver kinase B1 (LKB1) [31], directly regulates the proliferation, differentiation, and functional phenotypes of T cells [32,33]. However, the potential function of LKB1 on T cell differentiation and activity is contradictory to the effects of metformin discussed above. Although LKB1 may be highly involved in promoting thymocyte maturation and T cell proliferation, it was also suggested to negatively regulate peripheral T cell IL-17 and IFNγ production [33], which are two cytokines that are associated with the cytotoxic CD8^+^ T cell phenotype [34]. Similarly, knockout of the gene encoding LKB1 was observed to activate PI3K and STAT in T cells, which are pathways that are highly involved with T cells fate, which displayed concomitant increases in IL-6 and TNFα production [35]. These data would then suggest that metformin could instead repress an antitumorigenic phenotype in T cells if its action was through the induction of LKB1 activity. In contrast, the activation of LKB1 and downstream AMPK activity have been shown to promote the infiltration of CD8^+^ T cells into perivascular adipose depots in vivo in a murine model of hypertension [36]; whether this may apply to the adipocyte-rich omentum and peritoneum in the context of ovarian cancer metastasis has yet to be determined and remains an important mechanism to elucidate. 

The potential influence of AMPK-dependent metabolic reprogramming on T cell phenotypes has recently been reviewed [37], and as it is a direct substrate for LKB1 [38], may provide further insight into possible mechanisms by which metformin may regulate T cell differentiation and function. The exact relationship between metformin and LKB1, AMPK, and mTOR in T cell differentiation and function is unclear. Notably, metformin has not been shown to directly interact with either LKB1 or AMPK and may indeed have multiple intracellular targets in addition to its putative binding to mitochondrial complex I [39,40,41]. This may be especially relevant in its potential to directly bind and inhibit HMGB1 [40], a cytokine that is observed to regulate T cell activity and have a multitude of effects on other immune cells (reviewed in [42]). How the inhibition of HMGB1 by metformin could impact T cell function in the complex environment of the TME has yet to be evaluated. There is also some contention as to whether the doses exceeding the micromolar concentrations used in in vitro studies, including some of the studies discussed in this review, are relevant to the exposure of patients on the drug at doses indicated for its use in managing diabetes, which ranges from 500–2000 mg per day. However, it is suggested that treatment regimens often used in vivo are more closely representative of the concentration of the drug that would accumulate in target tissues, and which activate AMPK [43]. The possibility that metformin is acting independently of LKB1/AMPK in T cells in vivo may explain some of these discrepancies regarding the drug’s observed physiological activity and what has been demonstrated using genetic mouse models directly targeting the LKB1/AMPK axis. In addition to its possible effects on multiple cell types within the TME, a further complication arises in the systemic effects of metformin that may impact T cell function, namely, its antihyperglycemic effects through enhanced insulin sensitivity, which could impact glucose-dependent T cell differentiation and activity [44,45]. Thus, a more comprehensive understanding of the exact molecular mechanisms by which metformin regulates T cell development and functions in vivo, or in physiologically relevant in vitro models of the TME, are specifically required in order to properly assess the direct effects of the drug on T cell activity in the TME.

### 2.3. Metformin and Myeloid-Derived Suppressor Cells (MDSCs)—An Overview

Although direct evidence identifying the effects of metformin on T cell infiltration and immunoreactive phenotypes in HGSOC has yet to be conducted, indirect evidence suggests that metformin may inhibit an immunosuppressive microenvironment in OvCa through the inactivation of MDSCs. MDSCs are proposed to target a number of immune cells, including T lymphocytes, which can result in decreased responsivity to antigens or other factors (reviewed in [46]). A recent prospective epidemiological study indicated that high levels of MDSCs in the plasma could serve as a better predictor of malignant OvCa than the CTC/Treg ratio mentioned above [47]. Whether this may relate to patient mortality is unclear; however, in vivo data strongly support that an elevated number of MDSCs in the TME inhibits immune reactivity while increasing the onset of ascites formation and decreasing survival [48,49]. Among many features of MDSCs, the enzymatic activity of CD39 and CD73 have been shown to be a potent mechanism of immunosuppression [50]. While earlier studies had shown that OvCa tumor cells with increased CD73 enzymatic activity can dampen T cell sensitivity [51], only recently has it been shown that its activity on MDSCs can directly suppress T cell function in the TME of OvCa.

**Figure 2:**
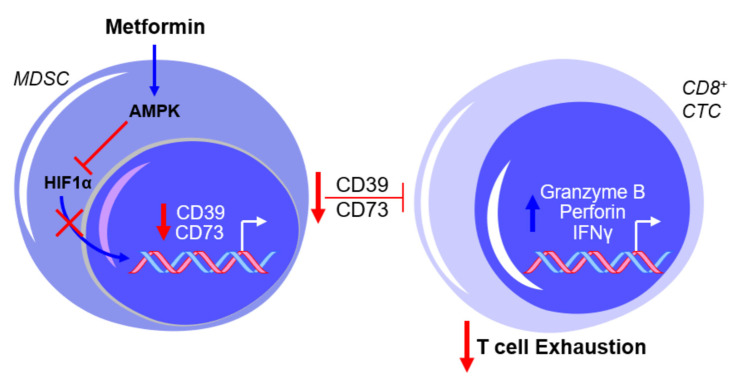
Metformin prevents myeloid-derived suppressor cell (MDSC)-mediated T cell exhaustion by downregulating CD39 and CD73 expression. The exposure of MDSCs to metformin activates AMPK and inhibits hypoxia-inducible factor 1-alpha (HIF1α) expression, thereby inhibiting the HIF1α-mediated transcriptional upregulation of CD39 and CD73 and subsequently suppressing cytotoxic CD8^+^ T lymphocytes (CTCs). This results in a net effect of reduced T cell exhaustion. Blue arrows indicate upregulation/activation, red arrows indicate downregulation/inactivation and red T symbols indicate inhibition.

In a study by Bin Zhang’s laboratory [52], it was shown that metformin inhibited the immunosuppressive activity of MDSCs through the increased activation of AMPK and decreased expression of hypoxia-inducible factor 1α (HIF1α), which resulted in the downregulation of CD39 and CD73 in MDSCs. Interestingly, the elevation of HIF1α was observed in the MDSCs derived from OvCa patients when compared to healthy controls. Whether AMPK and HIF1α are necessary for metformin’s effect on CD39 and CD73 expression and enzymatic function remains unclear. The use of compound C, an AMPK inhibitor, and CoCl_2_, an HIF1α activator, warrants caution with the interpretation of the results mentioned above due to the non-specificity of both drugs in their intracellular activity (e.g., [53,54]). However, these findings are consistent with the reported inhibition in MDSC function by AMPK activation and the proposed involvement of AMPK in the metabolic regulation of MDSC activity [55,56], as well as the putative transcriptional regulation of CD39 and CD73 by HIF1α [57,58]. Further testing by Li et al. showed that metformin treatment increased the production of granzyme B, perforin, and IFNγ by CD8^+^ T cells in vitro and in vivo, which was associated with decreased activity of immunosuppressive MDSCs resulting from the downregulation of CD39 and CD73 [52], as illustrated in Figure 2.

### 2.4. Metformin and MDSCs—Clinical Implications and Limitations

To complement their preclinical findings, the level of expression of CD39 and CD73 in MDSCs, as well as CD8^+^ T cell function, was measured in patients with type 2 diabetes mellitus (T2DM) on metformin. Consistent with their in vitro and in vivo data, metformin treatment significantly decreased CD39 and CD73 expression in MDSCs but increased CD8^+^ T cell function, as measured using granzyme B production. Intriguingly, these effects were observed in patients with diabetes before and after metformin treatment using a pairwise comparison (e.g., comparing the expression levels within patients following treatment) [52]. Presumably, these measurements were taken at the beginning and end of the 2-year prospective study; however, the exact duration and dosage of treatment is not specified. Nonetheless, the concept that 2 years (or less) of metformin treatment could have a profound impact on MDSC behavior in the TME is a strong indication that the drug may be useful in intervention treatment regimens, especially in patients that exhibit an immunosuppressed TME, or potentially as a chemopreventive agent in patients who are identified as being at high risk for developing an aggressive disease.

Taken together, this study by Li et al. supports a possible mechanism for metformin that prevents T cell exhaustion through inhibition by other immune cells, such as MDSCs, to maintain an immunoreactive TME. While promising, the in vitro data reached significance only at supraphysiologic levels of metformin at 2mM, which is markedly higher than the micromolar range (<100 µM) experimental equivalent to the clinically used doses of the drug [43]. It is unclear whether higher doses of the drug were necessary due, at least in part, to the high levels of glucose in the media during the metformin exposure, as its biological activity in vitro (e.g., AMPK activation) has been demonstrated to be significantly restricted by the elevated glucose observed in most cell culture media [59]. The ex vivo analysis of T cells from mice exposed to the drug also markedly exceeded clinically relevant concentrations at 10 mM; however, these data were strongly supported by the clinical findings. Further investigation on metformin’s modulation of the regulation of the immune milieu in the TME by MDSCs continues to be essential to determine whether metformin may be utilized as a therapeutic strategy to prevent immune evasion and the subsequent progression of HGSOC.

### 2.5. Metformin and Neutrophils—Neutrophil:Lymphocyte Ratio (NLR)

Cancer cells produce an inflammatory response, which results in increased levels of neutrophils and often decreased levels of functional lymphocytes. Neutrophils induce several cytokines, including vascular endothelial growth factor (VEGF), which aids in angiogenesis and tumor growth [60]. Lymphocytes, such as T lymphocytes, promote antitumor defenses through immune sensitivity and antibody- or cell-mediated apoptotic mechanisms [61]. An elevated NLR is an inflammatory marker that has been shown in studies to have a strong correlation with lower overall survival in patients with OvCa [62], with no established NLR value that confirms higher mortality. It is proposed that the effect of NLR on a patient’s survival rate depends on each patient’s baseline NLR, as well as their current therapy [62]. Further research is necessary to determine the extent to which the NLR value affects OvCa prognoses.

There is no clear indication that metformin may impact the NLR in patients with HGSOC. However, data in other inflammatory disease states suggest that the drug could potentially reduce NLR in several contexts. When looking at metformin in diabetes, a disease that has been observed to have an elevated NLR [63], it was observed that T2DM patients taking metformin had significantly decreased mean NLRs when compared to patients being treated with a sulfonylurea [64]. Patients with polycystic ovarian syndrome (PCOS), a disease associated with chronic inflammatory states and that has been associated with up to a threefold increased risk of developing OvCa [65,66], also often present with an elevated leukocyte count, which is largely due to the increased levels of circulating neutrophils [67,68]. When Ibanez and colleagues used metformin as a treatment compared to a placebo, metformin lessened the inflammatory response by significantly reducing the neutrophil count within 3 months of treatment [68]. Although these data were obtained from patients with hyperinsulinemic hyperandrogenism, a hallmark of PCOS in which patients display especially high neutrophil and leukocyte counts [68], it does support the possibility that metformin could prevent the increased neutrophil levels that are associated with aggressive HGSOC [62]. 

### 2.6. Metformin and Neutrophils—Neutrophil Extracellular Trap (NET)

An excess neutrophil count could predictably lead to the increased formation of NETs, which are the secretion of a network of fibers, including chromatin and proteins, that generally have microbicidal activity (reviewed in [69]). NET formation may also result in a specific form of cell death that is referred to as NETosis, in which destabilization of neutrophil membranes causes the release of a dense network of NETs that may induce persistent inflammation that is associated with several autoimmune diseases [69]. In a recent report by Honami Naora’s group [70] it was suggested that the increase in neutrophils migrating into the omentum facilitated the implantation of OvCa at this metastatic site. This resulted in the detrimental effects of OvCa-induced inflammatory signaling stimulating NET formation and subsequent NETosis. Notably, it was found that OvCa cells attached to NETs to metastasize into the omentum. If NET extrusion was inhibited by the genetic knockdown of a necessary enzyme for their formation (peptidylarginine deiminase 4 (PAD4)), omental metastasis was essentially ablated. There was no apparent effect of inhibited NET formation on the development of the primary ovarian tumor, suggesting that NETs promote the early dissemination and implantation of OvCa cells onto the omentum [70]. This study exemplifies the effectiveness of neutrophil depletion to prevent metastasis in early stage OvCa and strengthens the argument that the use of an agent, such as metformin, in a chemoprevention strategy in patients at high risk could potentially prevent early metastasis and improve patient outcomes. To this effect, studies by Menegazzo et al. identified the beneficial impact of metformin on NETs and NETosis [71]. In a randomized controlled trial, metformin was shown to significantly decrease the concentrations of NET biomarkers, such as dsDNA, histones, neutrophil elastase, and proteinase-3. In in vitro experiments, metformin was observed to halt NETosis through the inhibition of the PKC–NADPH oxidase (NOX) pathway. In addition to blunting NOX-dependent rupturing of the plasma membrane, metformin delayed the progression of the change in the nuclear shape of neutrophils, together slowing the release of NETs and ultimately NETosis [71]. 

**Figure 3:**
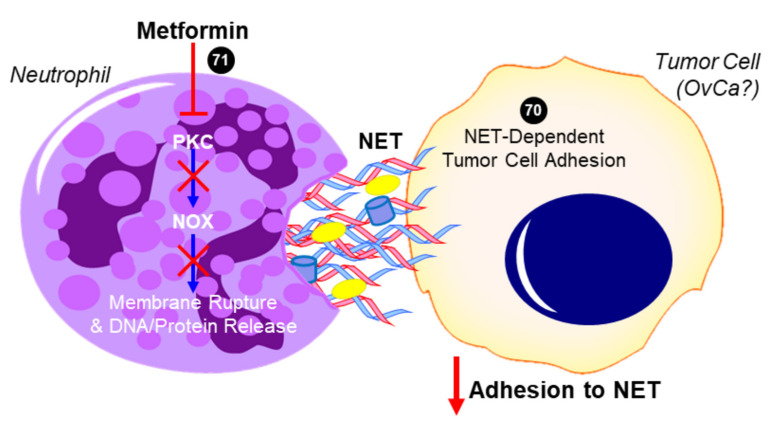
Proposed role of metformin in inhibiting neutrophil extracellular trap (NET)-induced tumor cell metastasis. In addition to decreasing neutrophil recruitment (not shown), metformin has been shown to inhibit protein kinase C beta II (PKCβII) translocation to the plasma membrane and prevent the downstream activation of NADPH oxidase (NOX) that would otherwise result in the expulsion of neutrophil extracellular trap (NET) components into the extracellular space [71]. Tumor cells have been shown to adhere to NETs, which contain DNA, histones, neutrophil elastase, and other proteins. In the case of ovarian cancer (OvCa), NET formation was especially critical to the ability of tumor cells to colonize the omentum in vivo, while having no effect on the primary tumor or other intra-abdominal sites [70]. The ability for metformin to inhibit this NET-dependent adhesion and colonization of the omentum has not yet been investigated but is a potential mechanism for its antitumor activity. Cited experiments are denoted by their respective reference numbers [70,71]. Blue arrows indicate activation, red arrows indicate inhibition, red T symbols indicate direct inactivation, and red X symbols indicate inhibition of this pathway by metformin.

While there is no direct evidence in HGSOC, these studies suggest that metformin’s anti-inflammatory effects may include a reduced neutrophil count, a subsequent decreased NLR, and the inhibition of NETosis (summarized in Figure 3), all of which have been identified as independent factors that are associated with decreased progression-free survival in OvCa. These data warrant further investigation of metformin and its regulation of NLR and NETosis in OvCa. By expanding in vivo studies to assess circulating neutrophils and leukocytes and fully characterizing the distribution of various immune cells within the primary tumor and secondary sites, as well as determining the impact of metformin on neutrophil functions (i.e., NETosis) in the omental TME, it will be possible to evaluate the effects of metformin on preventing a deleteriously pro-inflammatory microenvironment that promotes the metastasis of HGSOC.

### 2.7. Metformin and Macrophage Polarization

Patients with progressive ovarian carcinoma accumulate fluid in the peritoneal cavity, known as ascites. In the ascitic fluid, there are several types of cells present, including various leukocytes, and in particular an abundance of tumor-associated macrophages (TAM) [72]. The putative pro-tumorigenic M2 phenotype of TAMs often prevail over the anti-carcinogenic M1 TAMs within the ascitic fluid, which has been associated with a worse prognosis [73,74]. In assessing the effects of metformin on the macrophage infiltration of metastases, Wu et al. observed that metformin inhibited growth and metastasis to the liver, intestine, and lung, which was associated with decreased macrophage infiltration and tumor angiogenesis [75]. While TAMs have been observed to promote the metastatic potential of OvCa cells by upregulating angiogenesis [76], no direct evidence was provided in the study by Wu et al. It is notable that the in vitro assessment of metformin on OvCa proliferation, migration, and invasion was performed using doses up to 20 mM, which are markedly higher than the physiologic concentrations obtained in humans; however, the intervention treatment of the drug in in vivo xenograft models at 250 mg/kg does reflect clinically relevant concentrations of the drug used to treat T2DM [43]. Unfortunately, to date, no studies have experimentally evaluated the effect of metformin on macrophage polarization or function in HGSOC. A recent study of TAMs in breast cancer suggests that metformin may promote M2 polarization through the activation of AMPK in tumor cells by altering the signaling milieu to enhance the frequency of M2 phenotypes in the TME [77]; however, in the context of OvCa, this would potentially result in an immunosuppressive TME and consequent immune evasion by tumor cells (reviewed in [78]). Therefore, it is difficult to conclude what, if any, direct impact metformin has on TAM polarization in HGSOC and how this may affect tumor progression. Further studies using sophisticated models are required to dissect this potential relationship, and given the importance of TAMs on regulating immune sensitivity in the TME [78], this is a critical area of research that requires rapid and extensive advancement.

## 3. Considerations for Metformin on Immune Cell Metabolism

### 3.1. Impact of Metabolism on Immune Cell Differentiation and Function

As mentioned above (Section 2.2), one of the primary mechanisms of action for metformin is its inhibition of mitochondrial complex I, which results in alterations to cell metabolism, a process that generally results in the metabolic shift from OXPHOS to glycolysis that is referred to as the Warburg Effect [39]. The exact mechanisms underlying the regulation of immune cell function by metformin remains unclear; however, the ability for the balance between OXPHOS and glycolysis to regulate differentiation and the activity of T cells, MDSCs, neutrophils, and macrophages has been well described, and thus present one plausible mechanism by which metformin modulates immune cell phenotypes. This is especially important in the differentiation and activity of T cells, in which T lymphocytes associated with a CTC-like phenotype demonstrate predominantly glycolytic metabolism, whereas Treg and Tmem lymphocytes exhibit a preference for oxidative metabolism (reviewed in [79]). Conversely, lactate accumulation in the TME has been shown to directly inhibit CTC immune function, as well as promote MDSC activation and the consequent suppression of T cells [80,81]. The glycolytic shift has also been observed to promote neutrophil activation and NET formation, and macrophage polarization and their inflammatory activity is thought to be regulated largely by TCA intermediates, such as succinate downstream of glycolytic reprogramming [82,83]. Taken together, it is indeed likely that metformin exerts its beneficial effects on an immune-reactive TME, at least in part, through its promotion of glycolytic activity. However, an extensive investigation using in vivo models assessing immune cell metabolism and function is required to elucidate whether this is the predominant mechanism by which metformin modulates immune cell activity.

### 3.2. Metformin, AMPK, Glycolysis, and Ovarian Cancer

Metformin has been shown to induce AMPK activation and broadly alter the metabolic profile of a number of cell types in OvCa in vitro, including HGSOC tumor cells [84,85], fibroblasts [19], mesothelial cells [19], and MDSCs [52]. However, there is contention regarding the exact impact of AMPK itself on glycolytic metabolism. In both breast and lung cancer, it was separately shown that the inhibition of AMPK-dependent activation of PFK-2, the rate-limiting enzyme in glycolysis, prevented glycolysis in response to energy stress [86,87], which is consistent with early observations that phosphorylation of PFK-2 by AMPK was required for glycolytic activity in cardiomyocytes [88]. In contrast, AMPK activation was shown to suppress the tumor growth of lymphoma in vivo, which was associated with its inhibition of glycolysis [89]. These findings were consistent with the observation that, in leukemia cells under metabolic crisis, AMPK-dependent inhibition of mTOR was necessary to inhibit glycolysis and promote mitochondrial respiration [90]. 

This dualistic nature of AMPK in regulating glycolysis may thus depend on the cell type and tissue, as its intracellular activity is likely dependent on the proteomic composition (i.e., expression levels of mTOR, PFK2, etc.) and the intrinsic activity of its downstream kinases may determine the outcome of its activation. In light of these conflicting data regarding AMPK and the glycolytic shift, in order to elucidate how metformin may modulate immune cell metabolic reprogramming and consequent function, it is thus critical to either (1) perform a systematic analysis of the necessity of AMPK for metformin’s action using dominant-negative and other genetic constructs in vitro and in vivo [86], with the subsequent metabolomic profiling of immune cells; or (2) investigate alternative targets of metformin, such as those mentioned above, or possibly identify novel targets that may otherwise contribute to immune cell function. This is especially critical for considering the breadth of metformin’s regulation on a number of other metabolic and synthetic pathways in vitro [19], which further necessitates a holistic evaluation of the effects of metformin on immune cell metabolism. 

Lastly, it is important to consider that glycolysis mediated by GLUT1 expression and AMPK activation has been observed to markedly enhance OvCa tumor cell proliferation [91]. More recently, AMPK was shown to be indispensable for autophagy-mediated OvCa spheroid survival and proliferation [92], although whether and how it was influencing this phenotype has yet to be clearly defined. Although the majority of in vitro data using metformin in HGSOC and other OvCa cell lines demonstrate inhibited proliferation and colony formation [18,85], both of these pro-tumorigenic mechanisms could potentially be promoted by metformin through its well-defined upregulation of GLUT1 expression and AMPK activation. Thus, a more comprehensive understanding of the distinctive impact of the drug on different cell types within the TME in vivo and in situ will better inform its appropriate use in therapeutic strategies.

## 4. Conclusions

One of the difficulties in appreciating the potential capacity for metformin to restore immunoreactivity and minimize tumor development or progression is the inherently complicated relationship between the immune system, inflammation, and cancer. While inflammatory responses to tumor growth display a number of mechanisms that are known to stimulate tumor cell self-renewal, proliferation, and motility [93], the ability to induce tumor cell lysis through enhancing T cell cytotoxic activity [61] or by introducing the adoptive transfer of functional T cells [94] clearly supports the idea that the immune system, and subsequent inflammatory signaling, can also serve an anti-tumorigenic function. Similar to deregulated and persistent inflammation, that is, normophysiological inflammatory processes without successful resolution, this increased immune involvement may, on the other hand, confer progressive and potentially fulminant disease [95]. Thus, the balance between an anti- or pro-inflammatory immune milieu may determine the outcome of disease progression [96], and this apparent dichotomy in the role of the immune system in tumorigenesis remains an intensive focus of research. 

Too many patients succumb to the aggressive behavior of OvCa tumor cells. Recent advances in improving personalized medicine in HGSOC patients via the high-throughput screening of biopsies combining genomics, proteomics, and metabolomics is a notable potential approach in future clinical decision-making to improve survival, as using these strategies may allow for targeted therapy in patients to exploit therapeutic liabilities of tumors to PARP inhibitors (e.g., olaparib) or anti-angiogenic agents (e.g., bevacizumab) (reviewed in [97]). As metformin has been shown to potentially induce sensitivity to these agents and improve patient responses in several cancers [98,99,100,101], it is possible that the drug may be utilized as an adjuvant to improve the therapeutic response in specific subsets of HGSOC patients. Given the complexity of the TME in OvCa, coupling these approaches using laser capture microdissection may also further allow for the characterization of the stromal compartment and classification of the immune milieu to improve precision medicine. 

As seen comprehensively in the aforementioned studies, the addition of metformin in the immunosuppressed TME of patients from multiple cancers has shown to improve their immune cell function and sensitivity, which may relate to its ability to improve the outcome in highly aggressive diseases, such as HGSOC. While it has yet to be shown directly, the overlap in the role of immune cells across cancers implies that metformin may also be useful in restoring the immune-mediated inhibition of tumor development and progression in ovarian cancer. The capacity for metformin to promote cytotoxic CD8^+^ T lymphocytes, inhibit MDSC immunosuppressive function, decrease neutrophil recruitment and the NLR, and possibly repress TAM infiltration into the TME all suggest that the drug may enhance the immune sensitivity in HGSOC and thereby prevent metastasis. Further research is still crucial for examining additional mechanisms that metformin is involved with in HGSOC, as well as validating current results found from previous studies, before metformin can be confidently utilized as a new therapy in patients with HGSOC. 

It is important to note that much of the clinical data suggesting the benefit of metformin regarding patient outcomes are still predominantly in retrospective analyses, which makes it difficult to determine how the drug may be best utilized in therapeutic strategies. However, recent prospective clinical data do in fact support an improvement in the overall survival of patients with ovarian cancer treated with metformin [16], which in this study, were associated with the ability of metformin to both inhibit ALDH^+^/CD133^+^ cancer stem-like cells (CSCs) and increase CSC sensitivity to carboplatin ex vivo. Unfortunately, to date, only the study by Li et al. (Section 2.3 and Section 2.4 above) has investigated the effects of metformin on immune cell function in patients with ovarian cancer, and formal prospective clinical trials evaluating the impact of the drug on the immune TME have yet to be conducted. Given the potential for metformin to maintain an immune reactive TME, future clinical studies are needed to systematically assess the ability of the drug to facilitate immune cell-mediated inhibition of tumor development in HGSOC and other forms of ovarian cancer.

Currently, the preclinical data generally support the idea that metformin may prohibit an immunosuppressed TME and, if this is the case, could prevent the success of HGSOC cells in terms of metastasizing within the peritoneum. These data would then suggest that metformin may be most effective as a chemopreventive measure; however, with the nonspecific symptoms that are present in the early stages of disease development, it introduces another obstacle with regard to appropriately timing the initiation of metformin as a potential chemoprevention therapy. Moreover, the current prospective trials evaluating metformin in HGSOC and other ovarian carcinomas are intervention strategies in patients with stage II disease or above (Supplementary Table S1), which therefore would not be representative of the early neoplastic growth that is potentially derived from fallopian tube lesions and the subsequent serous tubal intraepithelial carcinomas [102], and thus may not detect whether the drug will be useful in preventing the early stages of development in some patients. How the immune milieu in the TME differs throughout these early stages and in the developed invasive or metastatic carcinomas has yet to be fully evaluated and may play an important role in the response to treatment with metformin.

Overall, these possibilities warrant extensive investigation of how metformin may impact the local and systemic immune responses to de novo tumorigenesis and the subsequent metastasis to distant tissues. Only by using clinically relevant doses in physiologically representative systems, such as in vitro organotypic or organoid model systems, as well as complementary in vivo approaches utilizing advanced cell-specific genetic techniques, will it become clear whether the drug may in fact suppress tumor development or progression. It may be specifically beneficial to focus on experimental strategies that reflect the epidemiological findings in which benefits were observed retrospectively in individuals on the medication to manage diabetes, and possibly for extended periods that may have preceded significant events during neoplastic growth and dissemination. While technically challenging, this could be accomplished through the chronic pretreatment of in vivo animal models prior to engraftment, or in vitro by utilizing continuous peristaltic systems for reliably consistent drug delivery coupled with recently developed organoid cultures that incorporate immune cells, as well as other stromal cells [103,104]. Further systematic characterization of the effects of metformin on immune cell function using these sophisticated methods will undoubtedly elucidate whether and how this drug may support an anti-tumorigenic immune environment to prevent disease progression in HGSOC and other subtypes of OvCa.

## Data Availability

No unpublished data was generated in the present study.

## Supplementary Materials

The following are available online at https://www.mdpi.com/1422-0067/22/2/867/s1.

## Abbreviations

AMPK	5′ AMP-activated protein kinase
CTC	cytotoxic (CD8^+^) T lymphocytes
CD4	cluster of differentiation 4
CD8	cluster of differentiation 8
CD25	cluster of differentiation 25
CD39	cluster of differentiation 39
CD73	cluster of differentiation 73
CoCl_2_	cobalt chloride
CTLA4	cytotoxic T lymphocyte antigen-4
Foxp3	forkhead box P3
GLUT1	glucose transporter 1
HGSOC	high-grade serous ovarian cancer
HIF1α	hypoxia-inducible factor 1-alpha
HMGB1	high mobility group box 1
IFNγ	interferon gamma
IL-2	interleukin 2
IL-6	interleukin 6
IL-10	interleukin 10
IL-17	interleukin 17
LKB1	liver kinase B1
MDSC	myeloid-derived suppressor cell
mTOR	mammalian target of rapamycin
NET	neutrophil extracellular trap
NF-κB	nuclear factor kappa B
NOX	NADPH oxidase
OvCa	ovarian cancer
OXPHOS	oxidative phosphorylation
PAD4	peptidylarginine deiminase 4
PARP	poly(ADP-ribose) polymerase
PD1	programmed death-1
PFK-2	phosphofructokinase 2
PKCβII	protein kinase C beta-II
T2DM	type 2 diabetes mellitus
TAM	tumor-associated macrophage
TIL	tumor-infiltrating lymphocyte
TIM3	T cell immunoglobulin and mucin-domain containing-3
TGF-β	tumor growth factor beta
TME	tumor microenvironment
Tmem	memory T cell
TNFα	tumor necrosis factor alpha
Treg	regulatory T cell
