# Supplementary material for: Possible Role of Metformin as an Immune Modulator in the Tumor Microenvironment of Ovarian Cancer
Source: Int J Mol Sci. 2021 Jan 16;22(2):867. doi: 10.3390/ijms22020867 (PMC7830067; doi:10.3390/ijms22020867)
Supplement: Supplementary file 1 [file ijms-22-00867-s001.pdf]

Study Title	Study Identifier	Trial Phase	Conditions	Interventions	Primary Outcome	Host Institution	Status
Study of Metformin With Carboplatin/Paclitaxel Chemotherapy in Patients With Advanced Ovarian Cancer (OVMET)	NCT02312661	Phase 1	Epithelial Ovarian Carcinoma (Stage 3+)	Paclitaxel/Carboplatin ± Metformin	ADEs within 2 years	University Medical Center Groningen	Completed
A Randomized WOO Study of Novel Therapeutic Agents in Women Triaged to Primary Surgery for EOC (IMPACT)	NCT03378297	Phase 1	Ovarian, Fallopian Tube or Primary Peritoneal Cancer (Stage 3+)	Metformin, Acetylsalicylic Acid, Olaparib or Letrozole prior to primary debulking surgery	Biomarker Expression within 3 months	Haukeland University Hospital	Recruiting
Study of Paclitaxel, Carboplatin and Oral Metformin in the Treatment of Advanced Stage Ovarian Carcinoma	NCT02437812	Phase 2	Epithelial Ovarian, Fallopian Tube or Primary Peritoneal Carcinoma (Stage 2+)	Paclitaxel/Carboplatin ± Metformin	PFS up to 1 year	Gynecologic Oncology Associates and University of North Carolina	Recruiting
Metformin and Chemotherapy in Treating Patients With Stage III-IV Ovarian, Fallopian Tube, or Primary Peritoneal Cancer	NCT02122185	Phase 2	Ovarian (Multiple Subtypes*), Fallopian Tube, Germ Cell or Primary Peritoneal Carcinoma (Recurrent or Stage 3+)	Paclitaxel/Carboplatin ± Metformin or Docetaxel/Carboplatin ± Metformin	PFS up to 2 years	University of Chicago	Recruiting
Evaluation of Metformin, Targeting Cancer Stem Cells for Prevention of Relapse in Gynecologic Patients	NCT01579812	Phase 2	Epithelial Ovarian, Fallopian Tube or Primary Peritoneal Carcinoma (Stage 2+)	Paclitaxel/Carboplatin ± Metformin	RFS up to 18 months	University of Michigan Rogel Cancer Center	Completed**
Study of the Safety, Tolerability and Efficacy of Metabolic Combination Treatments on Cancer	NCT02201381	Phase 3	Multiple Cancers including Ovarian Cancer	SOC ± Metformin, Atorvastatin, Doxycycline, or Mebendazole	OS up to 5 years	Health Clinics Limited	Not Yet Recruiting

Supplementary Table 1. Ongoing and Completed Trials including Ovarian Cancer Using Metformin in a Treatment Arm.
Status of each clinical trial is current as of January 2021, according to data from the NIH U.S. National Library of Medicine accessed at clinicaltrials.gov.
Abbreviations: ADEs – adverse drug events, PFS – progression free survival, RFS – recurrence free survival, OS – overall survival, SOC – standard of care.
*Subtypes include clear cell, endometrioid, mixed epithelial, serous, undifferentiated; study also included Brenner Tumor, malignant ascites and malignant pleural effusion
**Results for NCT01579812 have been reported and are available at PMID: 32369446 (Brown et al., JCI Insight 2020)
